# Prevalence and impact of tobacco use disorder on in-hospital mortality in patients hospitalized with non-group 1 pulmonary hypertension: a nationwide propensity score-matched analysis, 2019

**DOI:** 10.17179/excli2023-6409

**Published:** 2023-11-28

**Authors:** Rupak Desai, Zainab Gandhi, Saher taj Shiza, Akhil Jain, Hiren Koshiya, Bibi Alli-Ramsaroop, Agatha Olawunmi Akinsete, Eko Wilson, Pranathi Rudra, Mohan Sai Sunith Vegesna, Madiha Bari, Ankit Vyas, Bisharah Rizvi, Salim Surani

**Affiliations:** 1 Department of Cardiology, VA Medical Center, Atlanta, GA; 2 Department of Internal Medicine, Geisinger Wyoming Valley Medical Center, Wilkes Barre, PA/USA; 3 Department of Internal Medicine, Lincoln Hospital, Bronx, NY/USA; 4 Department of Internal Medicine, Mercy Catholic Medical Center, Philadelphia, PA/USA; 5 Department of Hematology/Oncology, Mayo Clinic, Jacksonville, FL/USA; 6 Department of Cardiology, Georgetown Public Hospital Corporation, Guyana; 7 Department of Medicine, Obafemi Awolowo University, Ilefe, Osun State, Nigeria; 8 Department of Medicine, Gandhi Medical College, Secunderabad, Telangana, India; 9 Department of Medicine, Jilin University, Changchun, China; 10 Department of Medicine, Nazareth Hospital, Philadelphia, PA, USA; 11 Department of Internal Medicine, Baptist Hospital of Southeast Texas, Beaumont, TX, USA; 12 Department of Pulmonary Medicine, UHS Southern California Medical Education Center, Temecula, CA/USA; 13 Department of Pulmonary and Critical Care, Texas A&M University, College Station, TX, USA

**Keywords:** pulmonary arterial hypertension, tobacco use disorder, smoker's paradox

## Abstract

Numerous studies indicated that patients with tobacco use disorder (TUD) are inversely associated with mortality in what is known as the smoker's paradox. However, limited studies have been conducted on the impact of TUD on the in-hospital mortality rates of patients with secondary pulmonary hypertension (PH, Non-Group 1 PH). Using the 2019 National Inpatient Sample, we identified PH and divided it into TUD and non-TUD to compare the comorbidities and in-hospital mortality between the two after 1:1 propensity-score matching. Of 1,129,440 PH hospitalizations, 12.1 % had TUD. After matching (n=133545, each group), TUD had lower median age (62 vs. 63), higher females (49 vs. 46.6 %), blacks (25.9 vs. 25.3 %), lower household income (40.8 vs. 32.7 %), Medicaid (22.4 vs. 14.8 %), non-elective (93.5 vs. 89.8 %), rural (9.3 vs. 6.7 %), urban non-teaching (17.2 vs 15.8 %) admissions. All CV comorbidities and other substance use were higher in TUD except CHF and valvular heart disease, TUD+ cohort and lower mortality (3.3 vs. 4.2 %, OR 0.78, p<0.001), higher routine discharges (53.8 vs. 51.3 %, p<0.001) and lower total charges ($47155 vs. 51909, p<0.001) than non-TUD. Although PH patients with TUD had a higher comorbidity burden, they had lower in-hospital mortality rates along with lower total charges of hospitalization, mandating real-world data to validate these results.

See also the Graphical abstract(Fig. 1).

## Introduction

Tobacco use disorder is a mental health condition characterized by nicotine dependence or tobacco addiction and uncontrollable use of these substances to avoid withdrawal via vaporizer pen, nicotine pen, and tobacco products. Several studies explored the association between tobacco use disorder (TUD)/ smoking and cardiovascular and pulmonary vascular diseases. Earlier studies have demonstrated that cigarette smoke, through multiple pathways, may induce activation of mitogen-activated protein kinase (MAPK) signal pathway and subsequent up-regulation of ET-1 and its receptors which then can cause receptor-mediated contraction, proliferation of pulmonary vascular smooth muscle cells, pulmonary vascular remodeling, and elevated pulmonary arterial pressure (Zhang and Xu, 2020[15]). As a result of altered blood arteries, pulmonary arterioles exhibit increased resistance, which is a hallmark of pulmonary arterial hypertension (PAH) (Emmons-Bell et al., 2022[6]). Smoking is a well-known risk factor for COPD, and research has revealed that chronic hypoxia causes systemic loss of the protein Hypoxia-inducible factor-1 (HIF-1), which has been proven to reduce pulmonary hypertension (Ball et al., 2014[3]). 

Tobacco use has also been implicated in the proliferation of pulmonary vascular smooth muscle through elevated nitric oxide and endothelin dysfunction (Seimetz et al., 2011[13]; Wright et al., 2004[14]) Reactions between nitric oxide and oxidants have been shown to increase pulmonary artery pressure, inherently leading to PAH. While tobacco use is implicated in the development of PAH (Kaneko et al., 1998[8]), limited data exist on the impact of TUD on the in-hospital outcomes of patients admitted with non-Group 1 Pulmonary hypertension/secondary pulmonary hypertension [non-group 1 PH].

## Materials and Methods

Using the weighted National Inpatient Sample dataset (AHRQ, 2019[1]) and previously validated ICD-10 codes, we identified admissions in patients with non-group 1 PH and divided it into TUD and non-TUD cohorts. In the United States, the NIS is a publicly accessible all-payer dataset that is part of the Healthcare Cost and Utilization Project (HCUP), sponsored by the Agency for Healthcare Research and Quality (AHRQ) (HCUP Database 2016-2017). Weighted NIS data is made up of about 35 million yearly in-hospital encounters from over 1000 non-federal acute care hospital centers in 45 states (excluding long-term acute care and rehabilitation centers). The weighted data is representative of over 95 % of community hospitalizations in the United States. The Institutional Review Board (IRB) approval was not mandatory as the NIS is public and does not reveal patients' identifiers. 

**Figure 2:**
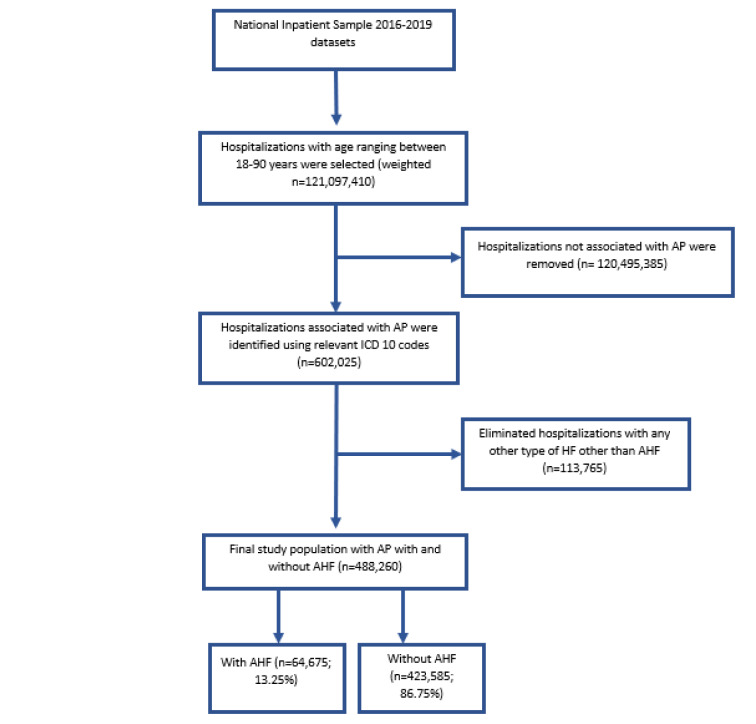
Study design and algorithm of patient selection ICD 10: International Classification of Diseases, Tenth Revision; AP: Aspiration Pneumonia; AHF: Acute Heart Failure

Propensity score matching (1:1) was performed, adjusting for age, sex, and race and 0.01 caliper width without replacement to obtain matched TUD+ vs. TUD- cohorts. We compared the two matched cohorts' baseline patient-level characteristics and preexisting comorbidities. The primary endpoint was the all-cause in-hospital mortality, and the secondary endpoint was healthcare resource utilization, including disposition of patients, hospital stay, and charges. Complex sample modules with IBM SPSS Statistics version 25.0 (IBM Corp., Armonk, NY, USA) and weighted data were used for all analyses. We used Pearson chi-square test for categorical measures and the Mann-Whitney U test for continuous variables. Multivariable regression analyses were performed controlling potential sociodemographic characteristics and pre-existing comorbid conditions. A two-tailed p-value of <0.05 was the threshold for statistical significance (Figure 2(Fig. 2)).

## Results

**Figure 3:**
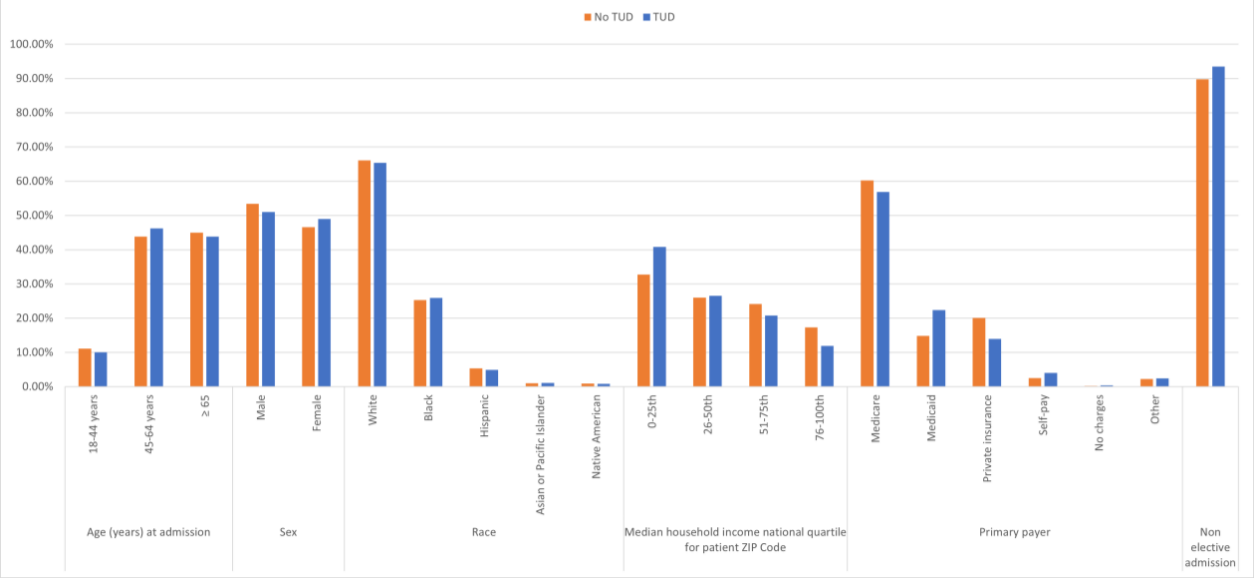
Differences in demographics of non-group 1 PH with vs without TUD

We identified 1,129,440 adult hospitalizations with non-group 1 PH in 2019; of those, 12.1 % had TUD. After matching (n=133545, each group), the TUD+ cohort consisted of younger (62 vs. 63), females (49 % vs. 46.6 %), blacks (25.9 % vs. 25.3 %), patients from lower household income (40.8 % vs. 32.7 %), Medicaid enrollees (22.4 % vs. 14.8 %) who were more often admitted non-electively (93.5 vs. 89.8 %) (Figure 3(Fig. 3)). Furthermore, we observed that the TUD+ cohort had more frequent admissions in rural (9.3 % vs. 6.7 %) and urban non-teaching (17.2 % vs 15.8 %) centers. The TUD+ cohort exhibited higher rates of comorbidities such as alcohol use (11.1 % vs. 3.9 %), depression (17.0 % vs. 14.35 %), AIDS (1.3 % vs. 1.0 %), peripheral vascular disease (13.4 % vs. 10.1 %), prior myocardial infarction (12.6 % vs. 10.5 %), prior coronary artery bypass grafting (8.7 % vs. 8.1 %), prior transient ischemic attack/stroke (8.6 % vs. 7.9 %), valvular disease (14.3 % vs. 14.2 %), liver disease (9.5 % vs. 8.4 %), drug abuse (13.1 % vs. 4.1 %), Cannabis use disorder (4.2 % vs. 1.3 %), chronic obstructive pulmonary disease (COPD) (62.4 % vs. 39.3 %), and Cancer (5.8 % vs. 6.8), in comparison to the TUD- cohort (Table 1(Tab. 1)).

**Figure 4:**
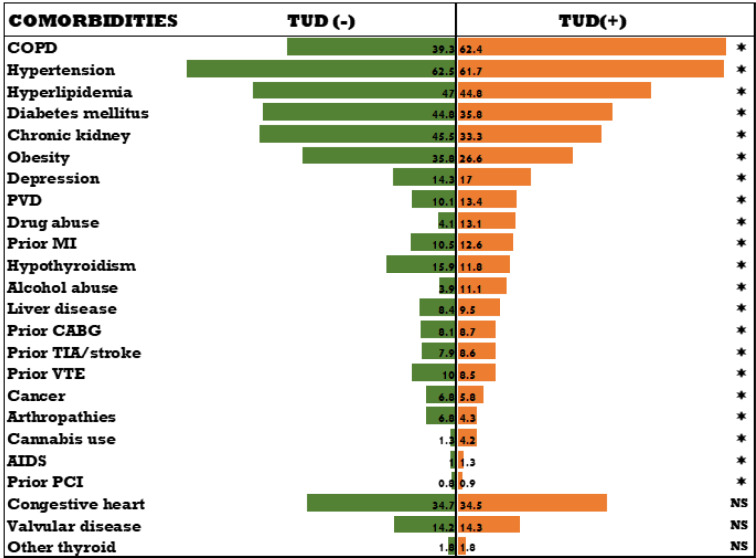
Differences in comorbidities of non-group 1 PH with vs without TUD COPD= chronic obstructive pulmonary disease; PVD= peripheral vascular disease; MI= myocardial infarction; CABG= coronary artery bypass grafting; TIA= transient ischemic attack; VTE= venous thromboembolism; AIDS= acquired immunodeficiency syndrome; PCI= percutaneous coronary intervention *: Significant; All values in the figure are in %.

However, The TUD- Cohort population demonstrated a greater prevalence of major cardiovascular comorbidities, including hypertension (62.5 % vs. 61.7 %), diabetes mellitus (44.8 % vs. 35.8 %), hyperlipidemia (47.0 % vs. 44.8 %), obesity (35.8 % vs. 26.6 %), as well as other comorbidities such as arthropathies (6.8 % vs. 4.3 %), prior venous thromboembolism (10 % vs. 8.5 %), chronic kidney disease (45.5 % vs. 33.3 %), and congestive heart failure (34.7 % vs. 34.5 %), hypothyroidism (15.9 % vs 11.8 %) (Figure 4(Fig. 4)) . (p:<0.001 for all, age, sex, race, median household income, insurance status, and all significant comorbidities except CHF and valvular disease and other thyroid disorders listed in Table 1(Tab. 1)). 

The TUD+ cohort had a lower rate and odds of mortality (3.3 % vs. 4.2 %, adjusted OR 0.78, p<0.001) vs. the TUD- cohort when adjusted for potential confounders. Furthermore, the TUD+ cohort often had routine discharges (53.8 % vs. 51.3 %, p<0.001), lower rate of transfers (16.9 % vs. 18.7 %, p<0.001), and less requirement of home healthcare (19.6 % vs. 21.2 %, p<0.001), which might have translated into lower total charges ($ 47155 vs. 51909, p<0.001) than TUD- cohort.

## Discussion

Smoking is a known major risk factor for cardiovascular diseases such as myocardial infarction (MI), peripheral vascular disease (PVD), and cerebrovascular diseases. Smoking remains a leading avoidable cause of death (Kondo et al., 2019[11]). We hypothesized the role of smoking on outcomes of pulmonary hypertension (non-group 1) hospitalization and mortality. The evidence supporting pathophysiology remains undetermined, like our study factors such as younger age and perhaps lower comorbidities were attributed to the findings (Aune et al., 2011[2]). Our study divided the non-group 1 PH cohort into TUD+ and TUD- groups. Our significant findings were TUD+ cohort was younger compared to TUD- cohort. Minor gender difference was noted in both groups. However, Keusch et al. showed gender disparity in smoking-related PH even when the active male and female smokers were similar. However, females had significantly higher secondhand smoke exposure contributing to the development of PH (Keusch et al., 2014[10]). 

Studies showed that metabolic syndrome is associated with a higher risk of all-cause mortality, cardiovascular risk, and CVD mortality (Guembe et al., 2020[7]). In our study, severe comorbidities such as prior MI, prior PCI, prior coronary artery bypass graft (CABG), chronic obstructive pulmonary disease (COPD), and prior transient ischemic attack (TIA)/Stroke were prevalent in the TUD+ cohort compared to TUD- cohort. But TUD- cohort had a higher prevalence of metabolic syndrome comorbidities such as HTN, DM, hyperlipidemia, and obesity. Studies so far have shown cardiovascular disease burden is directly correlated to the inflammatory status with increased vascular remodeling, which is noted in PH as well in the setting of tobacco use (Brassington et al., 2019[4]), the higher prevalence of cardiovascular risk, older age population in TUD- group could be the reason for higher all-cause mortality that supports smoker's paradox.

COPD is the main cause of pulmonary hypertension, which promotes the remodeling of pulmonary arterioles, and hypoxic vasoconstriction results in increased pulmonary blood pressure (Ball et al., 2014[3]; Chaouat et al., 2005[5]; Lam et al., 2009[12]). The TUD+ group has a higher prevalence of COPD (62.4 % vs 39.3 %). However, the impact of COPD on all-cause mortality compared to other morbidities in non-group 1 PH needs further investigation to validate the results. It stimulates the necessity for additional investigation, both in the context of research and a higher index in the non-group 1 PH cohort, to determine the outcome of this study. 

We discovered that the group TUD+ has lower all-cause mortality compared to TUD- cohort. Additionally, the length of stay was shorter in the TUD+ group, which reflected lower hospitalization costs than in the TUD- group which could be because the population is younger with overall less severe comorbidities. TUD + group had a higher rate of routine patient disposition (53.8 % vs. 51.3 %). The smoker's paradox of lower all-cause mortality, length of stay, and cost of hospitalization in the TUD+ group is not known in non-group 1 PH thus far. The only previous studies that have demonstrated smoker's paradox is in cardiovascular disease in the 1980s and then in 2011, with data supporting decreased in-hospital mortality for acute coronary syndrome in hospitalized patients (Aune et al., 2011[2]; Kelly et al., 1985[9]). Based on the results of our investigation, the smoker's paradox may be caused by differences in comorbidities and population ages, our study raises an important concern to understand the overall impact of smoking on outcomes of non-group 1 PH patients which is paradoxical to evidence thus far.

Like any study, ours has limitations. Inherent limitations of retrospective analysis including unable to control all confounders remains. NIS database has fundamental biases due to coding errors, administrative errors, or billing errors. Our two main groups of TUD+ and TUD- are based on patient's reporting which also creates a bias in the dataset, we were also unable to quantify smoking exposure, number of pack years. We were unable to differentiate between the types of non-group 1 PH due to NIS database billing and availability of sample size in each group. Given the retrospective observational analysis, only association can be concluded and the results of the smoker's paradox in the non-group 1 PH cohort need to be validated by additional prospective randomized data analysis, and further longitudinal studies are needed to determine which comorbidities are primarily responsible for the worse short-term outcome in the TUD group to form causative inferences. 

## Conclusion

Conclusively, non-group 1 PH patients with TUD+, despite having a higher comorbidity burden, however, they had lower in-hospital mortality rates along with lower healthcare resource utilization and lower hospital charges mandating prospective randomized data to validate these results of the “smoker's paradox” in better short-term outcomes in patients with non-group 1 PH and concomitant TUD.

## Notes

Rupak Desai and Zainab Gandhi contributed equally as first author.

## Acknowledgements

None.

## Figures and Tables

**Table 1:**
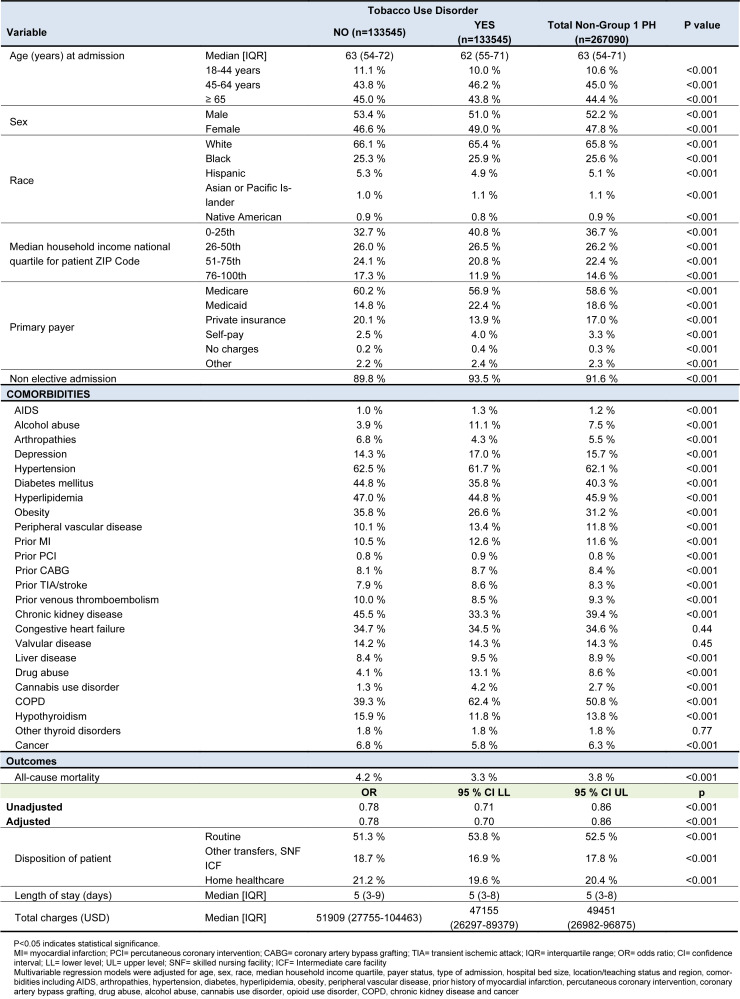
Adults hospitalized with secondary pulmonary hypertension [non-group- 1 PH] with vs. without tobacco use disorder (2019)

**Figure 1:**
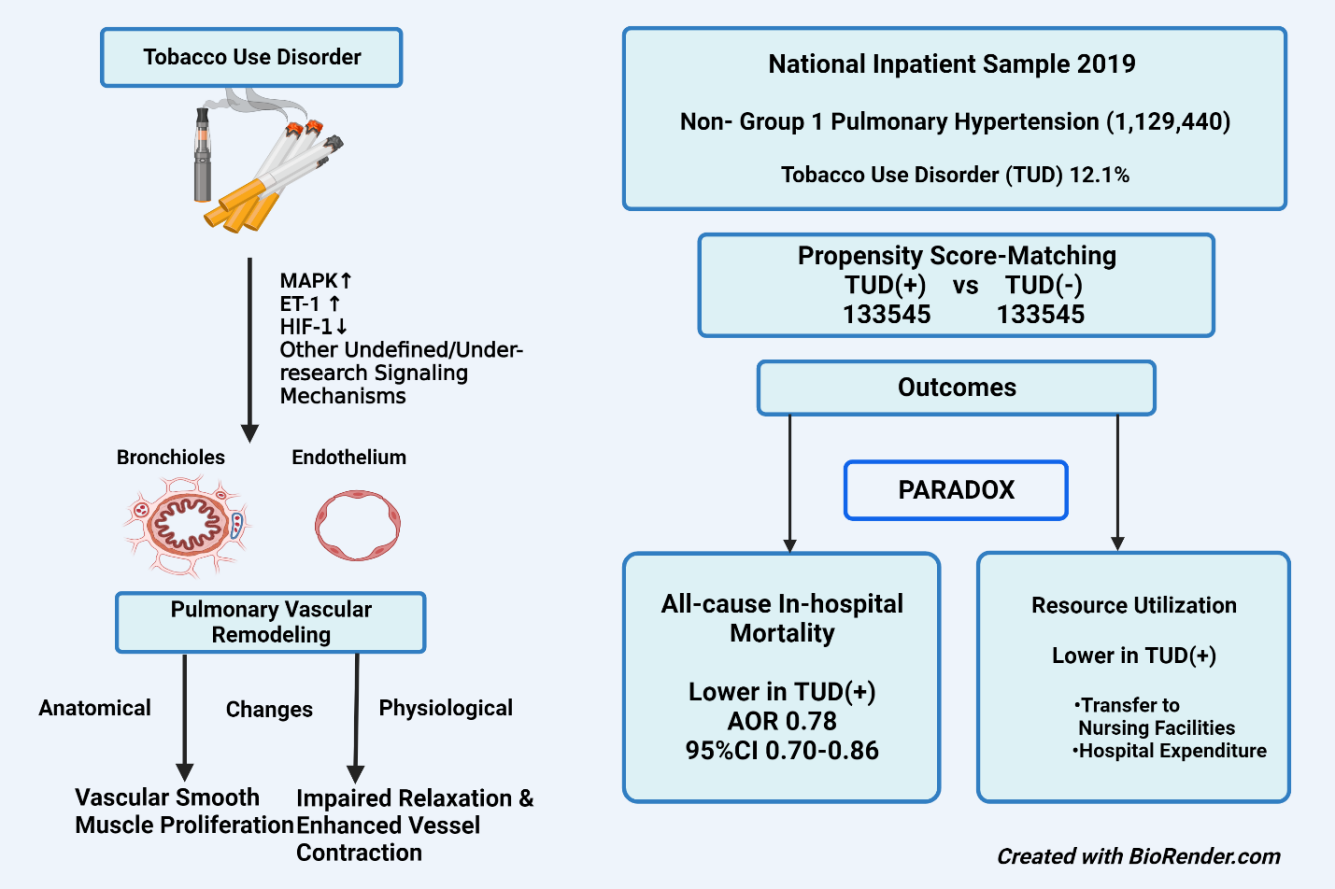
Graphical abstract

## References

[1] AHRQ, Agency for Healthcare Research and Quality (2012). HCUP National Inpatient Sample (NIS). Healthcare Cost and Utilization Project (HCUP).

[2] Aune E, Røislien J, Mathisen M, Thelle DS, Otterstad JE (2011). The “smoker’s paradox” in patients with acute coronary syndrome: a systematic review. BMC Med.

[3] Ball MK, Waypa GB, Mungai PT, Nielsen JM, Czech L, Dudley VJ (2014). Regulation of hypoxia-induced pulmonary hypertension by vascular smooth muscle hypoxia-inducible factor-1α. Am J Respir Crit Care Med.

[4] Brassington K, Selemidis S, Bozinovski S, Vlahos R (2019). New frontiers in the treatment of comorbid cardiovascular disease in chronic obstructive pulmonary disease. Clin Sci.

[5] Chaouat A, Bugnet AS, Kadaoui N, Schott R, Enache I, Ducoloné A (2005). Severe pulmonary hypertension and chronic obstructive pulmonary disease. Am J Respir Crit Care Med.

[6] Emmons‐Bell S, Johnson C, Boon‐Dooley A, Corris PA, Leary PJ, Rich S (2022). Prevalence, incidence, and survival of pulmonary arterial hypertension: A systematic review for the global burden of disease 2020 study. Pulm Circ.

[7] Guembe MJ, Fernandez-Lazaro CI, Sayon-Orea C, Toledo E, Moreno-Iribas C, RIVANA Study Investigators (2020). Risk for cardiovascular disease associated with metabolic syndrome and its components: a 13-year prospective study in the RIVANA cohort. Cardiovasc Diabetol.

[8] Kaneko FT, Arroliga AC, Dweik RA, Comhair SA, Laskowski D, Oppedisano R (1998). Biochemical reaction products of nitric oxide as quantitative markers of primary pulmonary hypertension. Am J Respir Crit Care Med.

[9] Kelly TL, Gilpin E, Ahnve S, Henning H, Ross J (1985). Smoking status at the time of acute myocardial infarction and subsequent prognosis. Am Heart J.

[10] Keusch S, Hildenbrand FF, Bollmann T, Halank M, Held M, Kaiser R (2014). Tobacco smoke exposure in pulmonary arterial and thromboembolic pulmonary hypertension. Respiration.

[11] Kondo T, Nakano Y, Adachi S, Murohara T (2019). Effects of tobacco smoking on cardiovascular disease. Circ J.

[12] Lam CSP, Roger VL, Rodeheffer RJ, Borlaug BA, Enders FT, Redfield MM (2009). Pulmonary hypertension in heart failure with preserved ejection fraction. J Am Coll Cardiol.

[13] Seimetz M, Parajuli N, Pichl A, Veit F, Kwapiszewska G, Weisel FC (2011). Inducible nos inhibition reverses tobacco-smoke-induced emphysema and pulmonary hypertension in mice. Cell.

[14] Wright JL, Tai H, Churg A (2004). Cigarette smoke induces persisting increases of vasoactive mediators in pulmonary arteries. Am J Respir Cell Mol Biol.

[15] Zhang Y, Xu CB (2020). The roles of endothelin and its receptors in cigarette smoke-associated pulmonary hypertension with chronic lung disease. Pathol Res Pract.

